# Imaging CAR-NK cells targeted to HER2 ovarian cancer with human sodium-iodide symporter-based positron emission tomography

**DOI:** 10.1007/s00259-024-06722-w

**Published:** 2024-05-09

**Authors:** Nourhan Shalaby, Ying Xia, John J Kelly, Rafael Sanchez-Pupo, Francisco Martinez, Matthew S Fox, Jonathan D Thiessen, Justin W Hicks, Timothy J Scholl, John A. Ronald

**Affiliations:** 1 Department of Medical Biophysics, Schulich School of Medicine and Dentistry, Western University, London, ON Canada; 2 Robarts Research Institute, Schulich School of Medicine and Dentistry, Western University, London, ON Canada; 3 Lawson Health Research Institute, London, ON Canada; 4 Lawson Cyclotron and Radiochemistry Facility, London, ON Canada; 5 Saint Joseph’s Health Care, London, ON Canada; 6 Ontario Institute for Cancer Research, London, ON Canada

**Keywords:** Positron emission tomography, Bioluminescence imaging, Cellular therapy, Natural killer (NK) cells, Chimeric antigen receptor (CAR), HER2

## Abstract

Chimeric antigen receptor (CAR) cell therapies utilize CARs to redirect immune cells towards cancer cells expressing specific antigens like human epidermal growth factor receptor 2 (HER2). Despite their potential, CAR T cell therapies exhibit variable response rates and adverse effects in some patients. Non-invasive molecular imaging can aid in predicting patient outcomes by tracking infused cells post-administration. CAR-T cells are typically autologous, increasing manufacturing complexity and costs. An alternative approach involves developing CAR natural killer (CAR-NK) cells as an off-the-shelf allogeneic product. In this study, we engineered HER2-targeted CAR-NK cells co-expressing the positron emission tomography (PET) reporter gene human sodium-iodide symporter (NIS) and assessed their therapeutic efficacy and PET imaging capability in a HER2 ovarian cancer mouse model.

NK-92 cells were genetically modified to express a HER2-targeted CAR, the bioluminescence imaging reporter Antares, and NIS. HER2-expressing ovarian cancer cells were engineered to express the bioluminescence reporter Firefly luciferase (Fluc). Co-culture experiments demonstrated significantly enhanced cytotoxicity of CAR-NK cells compared to naive NK cells. In vivo studies involving mice with Fluc-expressing tumors revealed that those treated with CAR-NK cells exhibited reduced tumor burden and prolonged survival compared to controls. Longitudinal bioluminescence imaging demonstrated stable signals from CAR-NK cells over time. PET imaging using the NIS-targeted tracer 18F-tetrafluoroborate ([^18^F]TFB) showed significantly higher PET signals in mice treated with NIS-expressing CAR-NK cells.

Overall, our study showcases the therapeutic potential of HER2-targeted CAR-NK cells in an aggressive ovarian cancer model and underscores the feasibility of using human-derived PET reporter gene imaging to monitor these cells non-invasively in patients.

## Introduction

Ovarian cancer is the most lethal gynecological cancer in women, with more than 65% of diagnosed women expected to die from the disease [1]. While surgical tumor debulking remains the main treatment for ovarian cancer, sometimes in combination with chemotherapy, more than 70% of patients recur and develop drug resistant tumors [2, 3]. New treatment strategies that have the potential to improve the survival of women with this disease are needed.

Advancements in the understanding of important biological drivers of cancer have led to a new and ever-expanding armamentarium of approved targeted therapies with many more in development [4]. Human epidermal growth factor receptor-2 (HER2), also known as HER2/erbB2, is a tumor-associated antigen that is over-expressed in ~ 10–20% of breast cancer cases, ~ 3% of cervical and uterine cancer cases [5], and ~ 27.6% of ovarian cancer cases [6]. HER2 initiates intracellular signalling pathways that promote tumor growth, survival and metastasis, and its overexpression is associated with poor clinical outcomes making this a promising and important therapeutic target [7, 8]. Trastuzumab (Herceptin) is a monoclonal therapeutic antibody directed against HER2 that has shown remarkable results against HER2 breast cancer [9]. However, it has not worked as well in HER2 ovarian cancer highlighting the need for alternative HER2-targeted therapies in the hope of improving patient outcome [10]. Importantly, a 2012 study showed that while conventional immunohistochemistry analysis showed HER2 overexpression in only 29% of evaluated tumor sites, using more sensitive methods emphasized that all human ovarian cancers express immunologically-detectable HER2 levels [11], highlighting the potential of HER2 as a targetable biomarker.

Chimeric antigen receptor (CAR) cell immunotherapies involve administering immune cells that have been genetically modified outside the body to target and kill cancer cells expressing a particular tumor-associated antigen. The extracellular domain of a CAR binds its target to initiate the activation of numerous intracellular signaling domains such as the T cell receptor (TCR)/CD3 complex and the co-stimulatory domains 41BB or CD28 [12, 13]. Unlike targeted therapies that treat the biomarker/antigen of interest (e.g.,. trastuzumab), the antigen in this case acts as a trigger to activate the CAR-T cells to promote cancer kill. CAR-T cells have shown transformative and even curative clinical results in patients with numerous types of hematological malignancies, and six CAR-T cell therapies have been approved in the United States. In addition, many new CAR designs are being explored for the treatment of solid tumors in both preclinical models and clinical trials [14, 15]. In the last 2 decades, many groups have developed HER2-directed CAR-T cells for the treatment of colorectal cancer [16], breast cancer [17], gastric cancer [18], sarcoma [19], glioblastoma [20], ovarian cancer [11], osteosarcoma [21], and medulloblastoma [22] in either patients or pre-clinical models. Early clinical trials with HER2 CAR-T cells, including an ongoing Phase I trial that includes ovarian cancer, have been performed [8, 19, 23–25].

Despite the remarkable clinical success, wider application of CAR-T cells has been partially hindered by life-threatening side effects such as cytokine release syndrome (CRS) and immune effector cell-associated neurotoxicity syndrome (ICANS) [26]. In addition, CAR-T cells have not demonstrated the same level of therapeutic efficacy against solid tumors as blood cancer, and many blood cancer patients can relapse following treatment or do not respond to CAR-T cell therapy at all [27]. Additionally, autologous cells are required as allogeneic T cells can trigger graft-vs-host disease (GvHD), which increases production complexity and time, and overall cost [28]. In the last decade, natural killer (NK) cells have become a promising alternative to T cells that have several benefits. NK cells have significant CAR-independent cancer cell killing potential through the recognition and binding of their activation receptors to cause the release of lytic granules [29–31]. NK cells also suppress GvHD, hypothesized to be attributed to the lysis of the recipient’s antigen presenting cells (APCs), and thus can be used as an allogenic source to make “off-the-shelf” or “universal” therapies [32–34]. Early studies in patients with B cell acute lymphoblastic leukemia with CAR-NK cells have shown promising efficacy without evidence of severe CRS, ICANS, or GvHD [35–40]. In 2022, there were 31 global clinical trials registered to address the safety and efficacy of CAR-NK cells in patients with hematological cancer [36]. One limitation to the widespread clinical use of primary NK cells is the difficulty to isolate, purify and expand them [32]. As an alternative, many groups have used NK-92 cells, an interleukin-2 (IL-2) -dependant human NK cell line, that can be easily expanded, engineered, and has demonstrated sustainable cytotoxicity against many cancer types [41–45].

In addition to making safer and more effective CAR therapies, many tools to better evaluate treatment response are being evaluated or developed as current tools are insufficient. For instance, traditional tumor imaging with computed tomography (CT) or ^18^F-fluorodeoxyglucose (^18^F-FDG) positron emission tomography (PET) has been used to demonstrate definitive treatment response or non-response in some patients, but these imaging tools can also show pseudoprogression where tumor size or metabolism increases even though the therapy is working [46]. To complement tumor imaging, ways to monitor the trafficking, infiltration and accumulation of CAR cells in tumors and elsewhere in the body could lead to earlier prediction of therapy response/non-response and side effects. Clinically, blood tests or single-site biopsies have been used to assess CAR-T cell dynamics but these methods are inadequate to predict response across multiple metastatic sites [46]. Alternatively, to track whole-body CAR cell distribution numerous groups have co-engineered CAR cells with PET reporter genes and assessed the ability to visualize these cells non-invasively in both animal models and patients [46–50]. Skovgard et al., developed CAR T cells with the PET reporter gene herpes simplex virus type 1 thymidine kinase (HSV1-tk) and correlated decreases in the bioluminescence imaging signal of luciferase-expressing mesothelin (MSLN) + tumors with increased PET signal of the CAR T cells [46]. Minn et al., co-engineered CD19 targeted-CAR T cells with a truncated prostate specific membrane antigen (tPSMA) for imaging with PET and reported an in vivo detection limit of 2,000 CAR T cells in mice [47]. Sellmyer et al., developed dihydrofolate reductase enzyme (eDHFR) from E.coli as a new PET reporter gene for CAR T cells [48]. Several groups have utilized the human sodium iodide symporter (NIS) to visualize CAR-T cells in mice [26, 51, 52]. NIS has several benefits as a clinically-relevant reporter gene as its human origin renders it non-immunogenic in patients, it has compatibility with many PET probes to allow for whole body PET imaging, and it can be paired with gamma or positron-emitting radioisotopes such as ^99m^Tc, ^123^I, ^124^I, ^131^I and ^18^F. Furthermore, NIS-expressing cells can also be treated with alpha- or beta- emitting radionuclides such as ^186^Re, ^188^Re, ^211^At, and therefore serve dual potential as a therapeutic/suicide gene [53–55]. Importantly, visualization of IL-13 receptor a2-targeted CAR-T cells co-expressing HSV1-tk has also been accomplished with PET in glioblastoma patients, establishing clinical feasibility of this approach [49, 50].

In this work, we developed trackable HER2-targeted CAR NK-92 cells co-expressing human NIS that were able to slow the progression of HER2 positive ovarian cancer in mice and could be non-invasively visualized using NIS-targeted PET.

## Materials and methods

### Cell lines

The HER2^+^ human ovarian cancer cell line, SKOV3-ip1, was a kind gift from Dr. Trevor Shepherd (University of Western Ontario) and cultured in McCoy’s 5a Medium Modified (ThermoFisher Scientific, 16600-082, Massachusetts, USA) supplemented with 10% fetal bovine serum (FBS) (ATCC® 30-2020™, Virginia, USA). NK-92 cells were purchased from American Type Culture Collection (ATCC® CRL-2407™, Virginia, USA). NK-92 cells were maintained in $\alpha$MEM (ThermoFisher Scientific, 12000-063, Massachusetts, USA,) supplemented with 1.5 g sodium bicarbonate (Sigma-Aldrich, S5761, Missouri, USA), 0.2 mM of Myo-inositol (Sigma-Aldrich, I-7508, Missouri, USA), 0.02 mM folic acid (Sigma-Aldrich, F-8758, Missouri, USA), 12.5% FBS (ATCC® 30-2020™, Virginia, USA), and 12.5% Horse Serum (ThermoFisher Scientific, 16,050,122, Massachusetts, USA). Complete media included 0.1 mM of 2-mercaptoethanol (ThermoFisher Scientific, MA, USA 21985-023) and 500 U/mL of recombinant human IL-2 (Sigma-Aldrich, I7908, Missouri, USA). All cells were kept in a humidified incubator with 5% CO_2_ at 37 °C and routinely confirmed to be mycoplasma-free using the MycoAlert mycoplasma detection kit (Lonza LT07-318, Basel, Switzerland).

### Cloning and lentiviral production

All cloning was performed using an In-Fusion HD Cloning kit (Takara Bio USA, Inc. CA, USA). A lentiviral transfer plasmid was made encoding the human elongation factor α promoter (pEF1α) driving an anti-HER2 CAR that uses a designed ankyrin repeat protein (DARPin) as the tumor-antigen targeting domain (a kind gift from Dr. Jonathan L. Bramson and described previously in [56]). Downstream of this CAR, the bioluminescence imaging (BLI) reporter gene Antares (Addgene plasmid # 74,279; http://n2t.net/addgene:74279; RRID: Addgene_74279) was cloned and separated by a T2A self-cleaving peptide sequence to make a pEF1α-HER2CAR-Antares transfer plasmid. A second transfer plasmid also driven by the pEF1 contained zsGreen (ZsG) followed by the NIS gene (Origene technologies, Inc. MD, USA NM_000453) separated by the T2A self-cleaving peptide sequence (pEF1α-ZsG-NIS). Finally, a previously made pEF1α-tdT-Fluc lentiviral transfer plasmid containing a tdTomato (tdT) [pUltra-Chili-Luc was a gift from Malcolm Moore (Addgene plasmid # 48,688; http://n2t.net/addgene:48688; RRID: Addgene_48688)] and the BLI reporter gene Firefly luciferase (Fluc) under the control of pEF1 was used (previously cloned and described in [57]).

For the pEF1-ZsG-NIS construct, lentivirus was produced by a commercial vendor at a titre of 10^8^ IFU/ml (Origene technologies, Inc. MA, USA: custom made). The other viruses were produced in house using each of the above transfer plasmids with the packaging and envelope plasmids pMDLg/pRRE, pRSV-Rev, and pMD2.G [pMDLg/pRRE was a gift from Didier Trono (Addgene plasmid # 12,251; http://n2t.net/addgene:12251; RRID: Addgene_12251), pRSV-Rev was a gift from Didier Trono (Addgene plasmid # 12,253; http://n2t.net/addgene:12253; RRID: Addgene_12253), pMD2.G was a gift from Didier Trono (Addgene plasmid # 12,259; http://n2t.net/addgene:12259; RRID: Addgene_12259), respectively). Human embryonic kidney (HEK 293T; ATCC, Virginia, USA) cells were transfected with Lipofectamine 3000 (Thermo Fisher Scientific, MA, USA) in accordance with the manufacturer’s lentiviral production instructions (Thermo Fisher Scientific Inc., MA, USA). After 24–48 h, viral-containing supernatant was collected, filtered with a 0.45${\upmu }\text{m}$ filter and frozen at -80 $^\circ$C until use.

### Lentiviral transduction

SKOV3-ip1 cells were transduced with the pEF1-tdT-Fluc lentiviral vector using 8-µg/mL polybrene. Transduced cells were sorted for tdT using a FACSAria III fluorescence-activated cell sorter (BD Biosciences, Ontario, Canada) to obtain Fluc^+^tdT^+^SKOV3-ip1 cells with a purity of 98%.

NK-92 cells were either transduced with one or both the pEF1-HER2CAR-Antares and/or pEF1-ZsG-NIS lentiviral vectors at a multiplicity of infection of 70 and with 8 µg/mL of polybrene. NK cells transduced with EF1-HER2CAR-Antares virus were sorted for cyOFP1 fluorescence in Antares and cells transduced with the EF1-zsG-NIS virus were sorted for zsG fluorescence. The brightest 10% of engineered cells were collected with a purity of 93% for Antares^+^ CAR NK cells, 98% for NIS^+^ NK cells (No CAR), and 98% for NIS^+^ Antares^+^ CAR NK cells.

### HER2 analysis

For assessment of HER2 expression, SKOV3-ip1 cells were stained using an Alexa Fluor 488 anti-human CD340 (ErbB2/HER2; 5 µl per million cells in 100 µl staining volume; BioLegend California, USA 324,410) prior to flow cytometric analysis using a FACS flow cytometer (BD FACSCanto™ Biosciences).

### Cytotoxicity assays

To evaluate immune cell cytotoxicity, Fluc^+^tdT^+^SKOV3-ip1 cells were cultured alone or with naïve NK cells, NIS^+^ NK cells, Antares^+^ CAR NK cells, or NIS^+^ Antares^+^ CAR NK cells at different effector: target (E: T) ratios. A 1:1 of effector to target ratio used 2 × 10^5^ cells of each cell type, and for different E: T ratios (2:1, 5:1), the number of effector NK cells was increased accordingly. After 24 h of co-culturing, Fluc BLI was performed to assess cancer cell viability by adding 150 µg/mL D-luciferin to each well and imaging plates on an IVIS Lumina XRMS scanner (PerkinElmer, MA, USA). Regions of interest were drawn over each well using LivingImage 4.5.2 software (PerkinElmer, Massachusetts, USA) and average radiance (p/s/cm^2^/sr) at peak signal was used.

Time-lapse fluorescence microscopy of Fluc^+^tdT^+^SKOV3-ip1 cells co-cultured with either NIS^+^ NK or NIS^+^ Antares^+^ CAR NK cells at a E: T of 2:1 was performed on a CytoSMART Lux3 FL incubator microscope (CytoSMART Technologies BV, AZ Eindhoven, Netherlands). Each image had an exposure time of 1020 ms, gain of 45, and intensity of 36%, and were acquired every 15 min for 36 h. The number of tdT-positive cancer cells over time were measured using the CytoSMART software.

### Animal model

Animals were cared for in accordance with the standards of the Canadian Council on Animal Care, and experiments were conducted as specified in our approved animal use protocol (AUP 2020-025). Fluc^+^tdT^+^SKOV3-ip1 cells (10^5^ in 100 µL) were injected intraperitoneally into immunocompromised female NOD scid gamma (NSG) mice and tumor progression was monitored with Fluc BLI as described below. Mice were administered intraperitoneally on days 8, 11 and 14 post cancer cell inoculation with either PBS (sham; *n* = 4), 1.5 × 10^7^ of naïve NK cells (*n* = 4), or 1.5 × 10^7^ NIS^+^ Antares^+^ CAR NK cells (*n* = 6). Following NK cell delivery, all mice received daily intraperitoneal injections of interleukin-2 (IL-2; 12,500 IU) except on treatment days 8, 11, and 14 and up until day 35.

### Fluc BLI of tumor burden

Mice were anesthetized with 2% isoflurane, injected intraperitoneally with 100 µl? D-luciferin (150 µg/mL) (Sigma Aldrich, Missouri, USA 808,350) and imaged with an IVIS Lumina XRMS In Vivo Imaging System (PerkinElmer, Massachusetts, USA). Images with auto exposure times were captured for 30 min, with a field of view of 12 cm. Regions of interest were drawn manually around the entire mouse body using the LivingImage software (PerkinElmer, Massachusetts, USA) to measure the average radiance (p/s/cm^2^/sr). Peak average radiance observed during the 30 min scan time was used for data analysis.

### Antares BLI of NIS^+^ Antares^+^ CAR NK cells

BLI of mice receiving NIS^+^ Antares^+^ CAR NK cells was performed over 30 min following intraperitoneal injections of the Antares substrate fluoroflurimazine (FFZ) as previously described (50 µL of stock solution diluted 50x in PBS; 50 µL injected; FFZ was kindly gifted by Promega, Wisconsin, USA) [58]. Regions of interest were drawn manually around the entire mouse body and the peak average radiance (p/s/cm^2^/sr) over the 30-minute imaging window was determined.

### [^18^F]TFB PET

PET imaging was performed to evaluate the ability of visualize NIS^+^ Antares^+^ CAR NK cells. Mice were anesthetized with 2% isoflurane, injected with 10–15 MBq of [^18^F]TFB in 100–150 $\mu$L and imaged with a Siemens Inveon™ microPET system (Siemens Medical Solutions USA, Inc.). Animal breathing rate and body temperature were monitored and kept between 40 and 70 bpm and at 37 °C, respectively, using a custom-made animal holder that allowed for simultaneous multi-mouse imaging. Static PET images were acquired 30 min post [^18^F]TFB tail vein injection. Images were reconstructed using ordered subset expectation maximization (OSEM) 2D. Quantification of PET signal was performed by manual segmentation of ROIs using Horos Project software v3.3.6. Maximum Activity Projections (MAPs). Standard Uptake Value (SUV) was calculated with the below equation:

$$SUV \left(\frac{g}{mL}\right)=\frac{Pixel value \left(\frac{Bq}{mL}\right)*weight \left(kg\right)}{Dose \left(Bq\right)}*1000 \left(\frac{g}{kg}\right)$$

### Peritoneal lavage

Mice were euthanized by cervical dislocation and the peritoneal cavity was flushed with 1 mL of PBS using a 27-gauge syringe, followed by aspiration of the peritoneal fluid to assess for cellular composition. The aspirate was strained with a 70 μm nylon strainer, spun down at 200 rcf for 10 min, and resuspended in 1 mL of PBS buffer solution containing 2% FBS, 1 µM EDTA, and 25 µM HEPES. Cell suspensions were assessed for the presence of Fluc^+^tdT^+^SKOV3-ip1 and NIS^+^ Antares^+^ CAR NK cells using a FACSCanto flow cytometer and FlowJo v10 software (BD Biosciences, Ontario, Canada).

### Histology and microscopy

Tumor masses within the peritoneal space, as well as the ovaries, were collected and fixed in 4% paraformaldehyde for 24 h. Half the tumor masses and one ovary from each mouse were embedded in paraffin, sectioned, and stained with hematoxylin-eosin. The remaining ovary and the other half of tumor tissue were immersed in increasing concentrations (10, 20 and 30%) of sucrose solutions for freezing. Frozen tissues were then cryosectioned and fluorescence microscopy was performed to visualize NIS^+^ Antares^+^ CAR NK cells (also expressing ZsG). Both bright-field and fluorescence images were acquired with a Revolve fluorescence microscope (Echo Microscopes, California, USA).

### Statistical analysis

Two-way repeated measures Analysis Of Variance (ANOVA) with multiple comparisons were used to compare Fluc and Antares BLI radiance over time between mouse cohorts. Mouse survival data were displayed on a Kaplan-Meier curve and a logrank Mantel-Cox test was used to compare survival between cohorts. For PET data analysis, an unpaired t test (assuming Welch correction) was used to compare the SUV values between cohorts. Statistical analysis was performed with GraphPad Prism Software (Version 7.00 for Mac OS X, GraphPad Software Inc., La Jolla California USA, www.graphpad.com). All data are expressed as mean ± standard deviation of at least three independent experiments and a p-value less than 0.05 was considered statistically significant.

## Results

### Genetic constructs and *in-vitro* validation

**Fig. 1:**
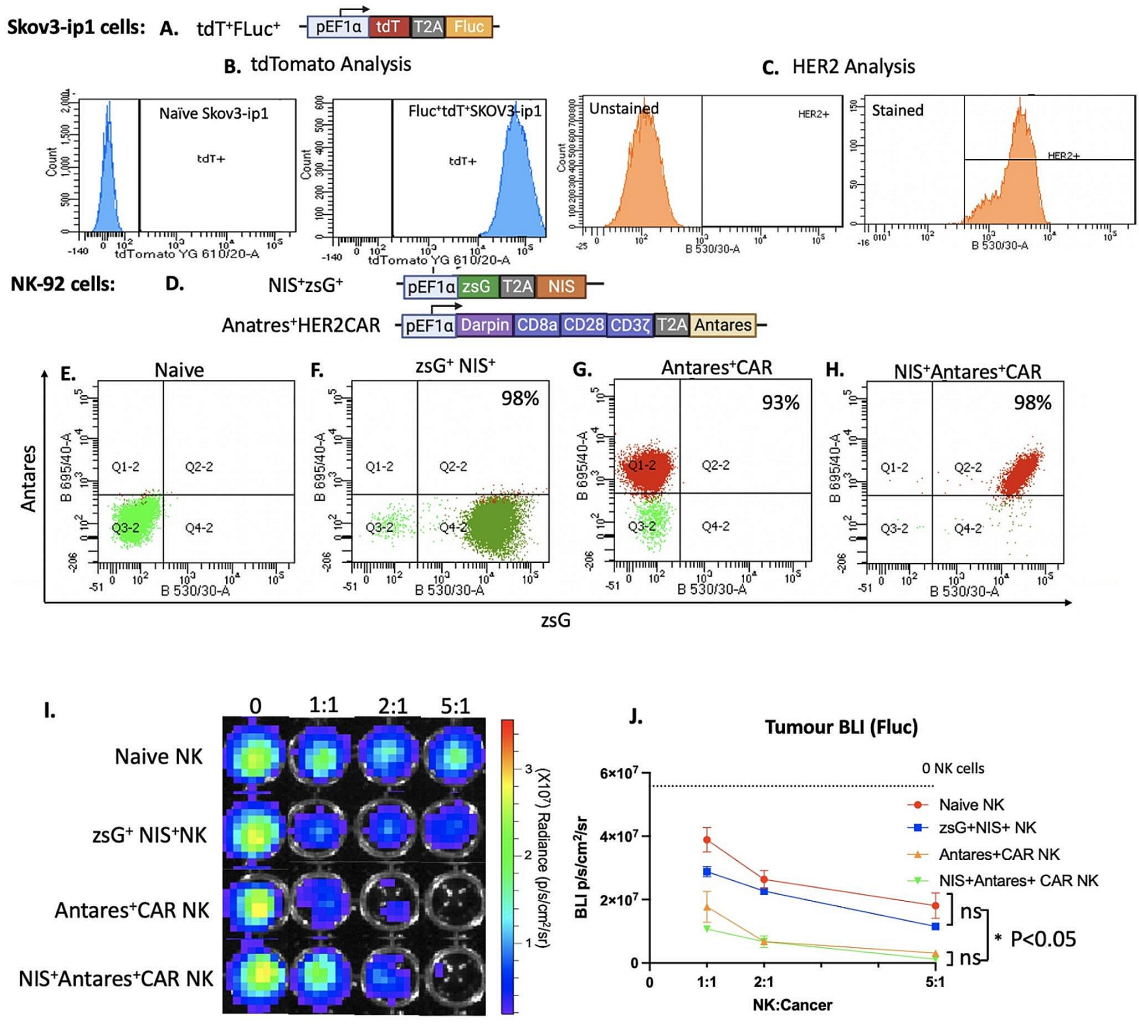
(**A**) Lentiviral construct used to engineer SKOV3-ip1 ovarian cancer cells containing the human elongation factor 1 alpha promoter (pEF1a) to drive expression of tdTomato (tdT) and Firefly luciferase (Fluc) separated by a T2A peptide sequence. (**B**) Flow plots tdTomato expression in naïve SKOV3-ip1 and Fluc^+^tdT^+^SKOV3-ip1. (**C**) Flow plots showing Fluc^+^tdT^+^SKOV3-ip1 cells with or without HER2 immunostaining. (**D**) Lentiviral constructs used to engineer NK-92 cells. The first plasmid construct containing zsG and NIS gene separated by a T2A upstream of a pEF1a promoter was used for NK-92 transduction. The second plasmid contained a pEF1a promoter upstream of a HER2 CAR gene (containing DARPIN, CD8a, CD28, CD3ζ components), in addition to Antares, was used for the sequential transduction of NK-92. (**E-H**) Flow plots confirming the expression of zsG and/or Antares in NK-92 cells engineered with one or both constructs in D. **I**) BLI images (**I**) and analysis (**J**) of cytotoxicity assays upon co-culturing of Fluc^+^tdT^+^SKOV3-ip1 with different NK cell phenotypes at various effector to target ratios

The lentiviral construct in Fig. 1A was used to make Fluc^+^ tdT^+^SKOV3-ip1 cells with a purity of 98% (Fig. 1B). Flow cytometry after immunostaining confirmed HER2 expression in SKOV3-ip1 cells (Fig. 1C). Figure 1D shows the lentiviral constructs used to engineer NK-92 cells. First, NK cells were transduced with either a lentivirus encoding zsG and NIS to make NIS^+^ NK cells (98% purity after sorting; Fig. 1F) or a HER2-CAR-Antares lentivirus to make Antares^+^ CAR NK cells (93% purity after sorting; Fig. 1G). A subset of NK-92 cells was transduced with both vectors to produce NIS^+^ Antares^+^ CAR NK cells (purity of 98% after sorting; Fig. 1H). Figure 1I and J show a BLI cytotoxicity assay using the four effector NK cell phenotypes against Fluc^+^tdT^+^SKOV3-ip1 cells at different effector to target ratios. Average BLI radiance (p/sec/cm^2^/sr) of cells demonstrated significantly higher cytotoxicity with CAR-expressing NK cells in comparison to non-CAR expressing NK cells at all E: T ratios (*p* < 0.05). No differences in cytotoxicity were observed between non-CAR expressing NK cells (naïve NK cells and NIS^+^ NK cells) or CAR-expressing NK cells with one or multiple reporter genes (Antares^+^ CAR NK cells and NIS^+^ Antares^+^ CAR NK cells).

### *In-vivo* inoculation and BLI tumour tracking

**Fig. 2:**
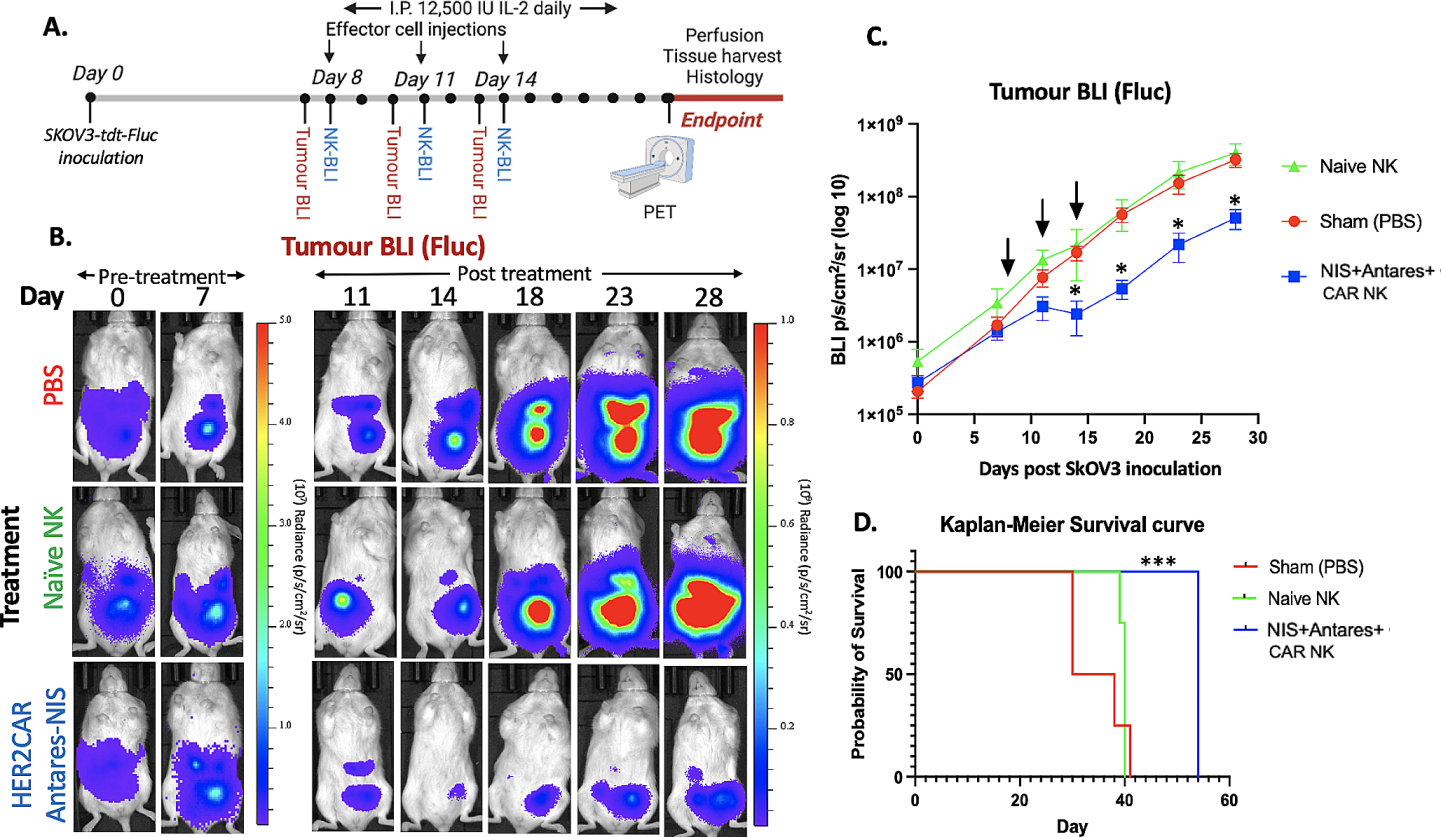
(**A**) Experimental design of mouse studies. (**B**) Fluc BLI images of tumor burden from a representative mouse from each cohort (PBS (*n* = 4), Naïve NK (*n* = 4) and NIS^+^ Antares^+^ CAR NK (*n* = 6)). (**C**) Average Fluc BLI radiance from all mice showed significantly lower tumor burden in mice receiving NIS^+^ Antares^+^ CAR NK cells compared to both other control cohorts at day 14, 18, 23 and 28 (*p < 0.05). (**D**) Kaplan-Meier curve analysis showed significantly increased survival in mice receiving NIS^+^ Antares^+^ CAR NK cells compared to mice receiving PBS or Naïve NK cells (***p < 0.001)

For *in-vivo* experiments, 10^5^ Fluc^+^tdT^+^SKOV3-ip1 cells were injected intraperitoneally into 14 NSG mice and tumor progression was monitored with Fluc BLI (Fig. 2A). One week post inoculation, mice received either intraperitoneal injections of PBS (*n* = 4), naïve NK cells (1.5 × 10^7^; *n* = 4), or NIS^+^ Antares^+^ CAR NK cells (1.5 × 10^7^; *n* = 6). These injections were repeated on days 11 and 14. Mice receiving any NK cells also received daily intraperitoneal injections of IL-2 (12,500 units) except for therapy days. As demonstrated by Fluc BLI signal from Fluc^+^tdT^+^SKOV3-ip1 cells, mice receiving PBS or naïve NK cell injections showed continuous and rapid growth of tumors (Fig. 2B). Mice receiving NIS^+^ Antares^+^ CAR NK cells showed significantly reduced average BLI signal compared to both control groups on days 14, 18, 23 and 28 (*p* < 0.05; Fig. 2C). Additionally, the NIS^+^ Antares^+^ CAR NK cell therapy resulted in a significantly higher survival compared to mice receiving either PBS or naïve NK cells (*p* < 0.001; Fig. 2D).

### Administration of NK cell therapies and tracking with PET

**Fig. 3:**
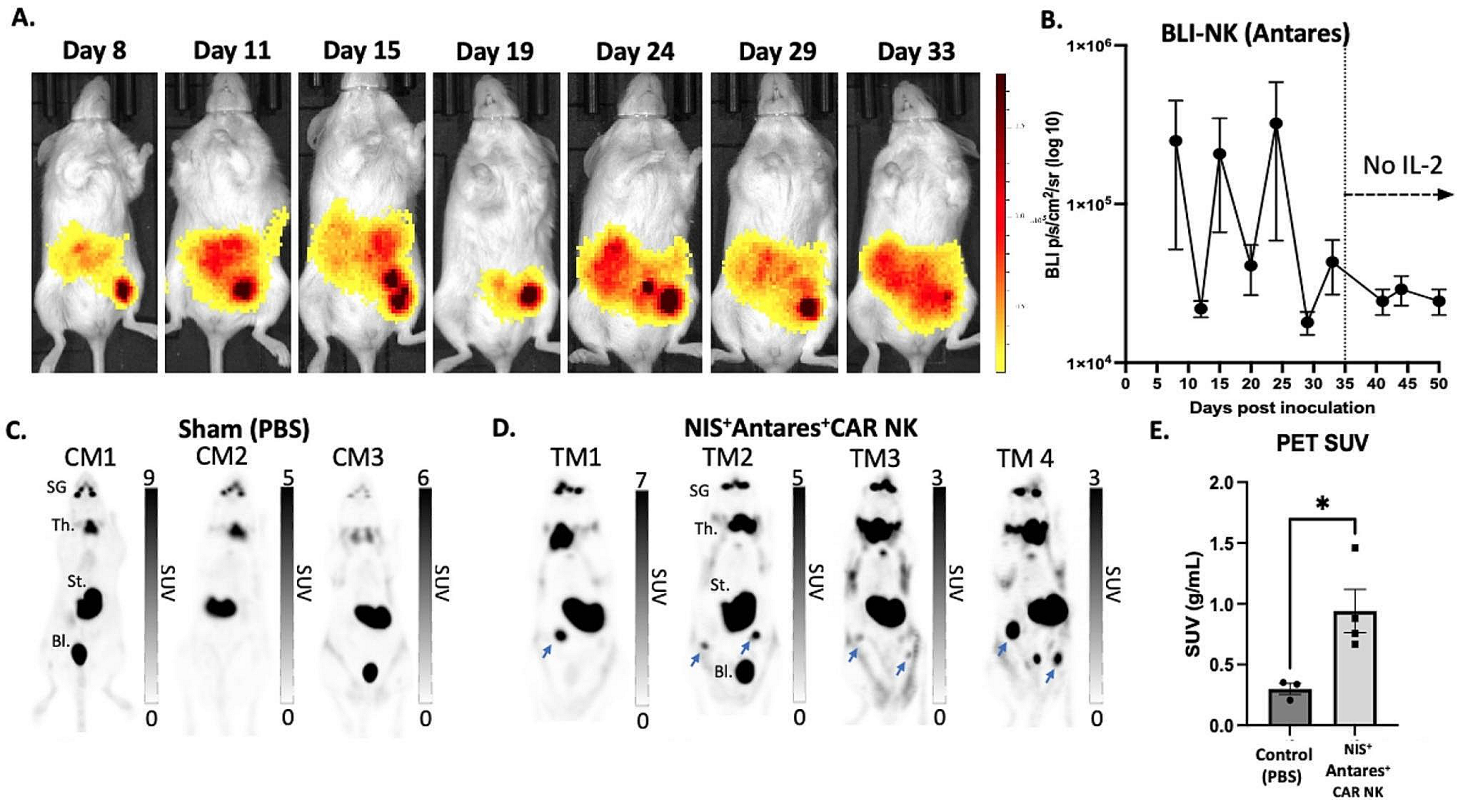
(**A**) Longitudinal Antares BLI images of a representative mouse receiving NIS^+^ Antares^+^ CAR NK cells. (**B**) Average radiance from Antares BLI images from all mice receiving NIS^+^ Antares^+^ CAR NK cells. Ceasing daily injections of IL-2 at day 35 resulted in stable Antares BLI signal from NIS^+^ Antares^+^ CAR NK cells. **C** and **D**) ^18^F[TFB] PET images of control mice (CM) receiving sham (PBS) injections and treated mice (TM) receiving NIS^+^ Antares^+^ CAR NK cells on day 27 post cancer cell inoculation. ^18^F[TFB] uptake is noted in NIS-expressing tissues and clearance routes (salivary glands (SG), thyroid (Th.), stomach (St.) and bladder (Bl.)) shows uptake of ^18^F[TFB]. Additional ^18^F[TFB] uptake is noted in the peritoneal of all mice receiving NIS^+^ Antares^+^ CAR NK cells. **E**) Significantly higher peritoneal PET signal (SUV) was measured in mice receiving NIS^+^ Antares^+^ CAR NK cells compared to mice receiving PBS injections (*p < 0.05)

Starting the day of NK cell administration (Day 8), mice receiving NIS^+^ Antares^+^ CAR NK cells were additionally imaged with Antares BLI twice per week (Fig. 3A). Antares BLI signal showed NIS^+^ Antares^+^ CAR NK cells consistently within the peritoneal space. High Antares signal was shown in similar areas with high Fluc signal, suggesting co-localization. Slight fluctuations in Antares BLI signal also corresponded to NIS^+^ Antares^+^ CAR NK cell injections. Daily injections of IL-2 to mice were halted on day 35 and the Antares BLI signal remained relatively stable thereafter until endpoint at day 50. On Day 27, mice receiving either NIS^+^ Antares^+^ CAR NK cells (*n* = 4) or PBS injections (*n* = 3) were imaged with PET using the NIS tracer ^18^F[TFB] (Fig. 3C & D). Maximum activity projections (MAPs) are shown where mice receiving PBS injections display uptake in endogenous tissues expressing NIS such as the salivary glands, thyroid, and stomach, as well as signal from the bladder due to the urinary clearance of the ^18^F[TFB] probe. In contrast, mice receiving NIS^+^ Antares^+^ CAR NK cells showed additional ^18^F[TFB] uptake in regions within the peritoneal cavity. Regions of interest (ROIs) were drawn over these areas in the NIS^+^ Antares^+^ CAR NK cell group, as well as general peritoneal ROIs for the PBS group, and analysis showed significantly higher peritoneal PET signal (SUV) in treated mice (Fig. 3E).

### Peritoneal lavage

**Fig. 4:**
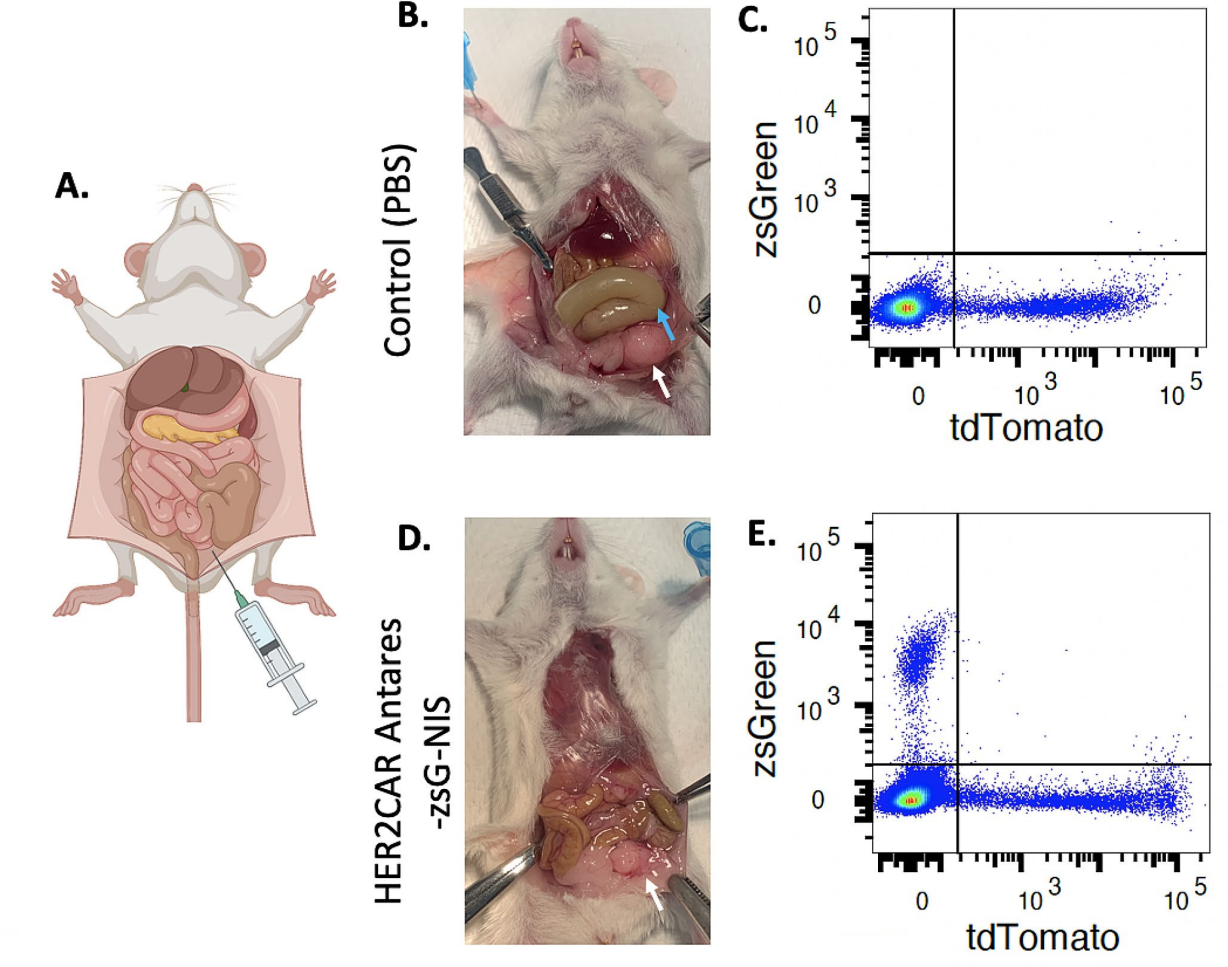
(**A**) Peritoneal lavages performed with PBS injections into the peritoneal space followed by aspiration to collect and assess cellular composition. (**B**) Control mouse receiving PBS injections showed larger cancer mass (white arrow) and ascites (blue arrow) within the peritoneal spaces. **D**) Mouse receiving the NIS^+^ Antares^+^ CAR NK cell therapy showed smaller tumor mass (white arrow) and no signs of ascites. **C** and **E**) Flow cytometry assessment of peritoneal lavage fluid showed tdTomato positive cells in both mice with additional zsGreen positive cells only in the mouse receiving the NIS^+^ Antares^+^ CAR NK cell therapy

On day 30, one control mouse and one mouse receiving NIS^+^ Antares^+^ CAR NK cells were sacrificed, and peritoneal lavages were performed to assess the cellular composition within the peritoneal cavity (Fig. 4A). Assessment of the cellular aspirate with flow cytometry revealed tdTomato positive cancer cells in both mice but zsGreen positive cells only in the mouse receiving NK cell therapy (Fig. 4C and E). Following lavage, laparotomies were performed for gross assessment of the presence of ascites and tumors. In a control mouse, ascites within the peritoneal space and a large tumor mass were present (Fig. 4B). In contrast, a mouse receiving NIS^+^ Antares^+^ CAR NK cells which showed minimal signs of ascites and had a visually smaller tumor lesion (Fig. 4D).

### Histology and fluorescence microscopy

**Fig. 5:**
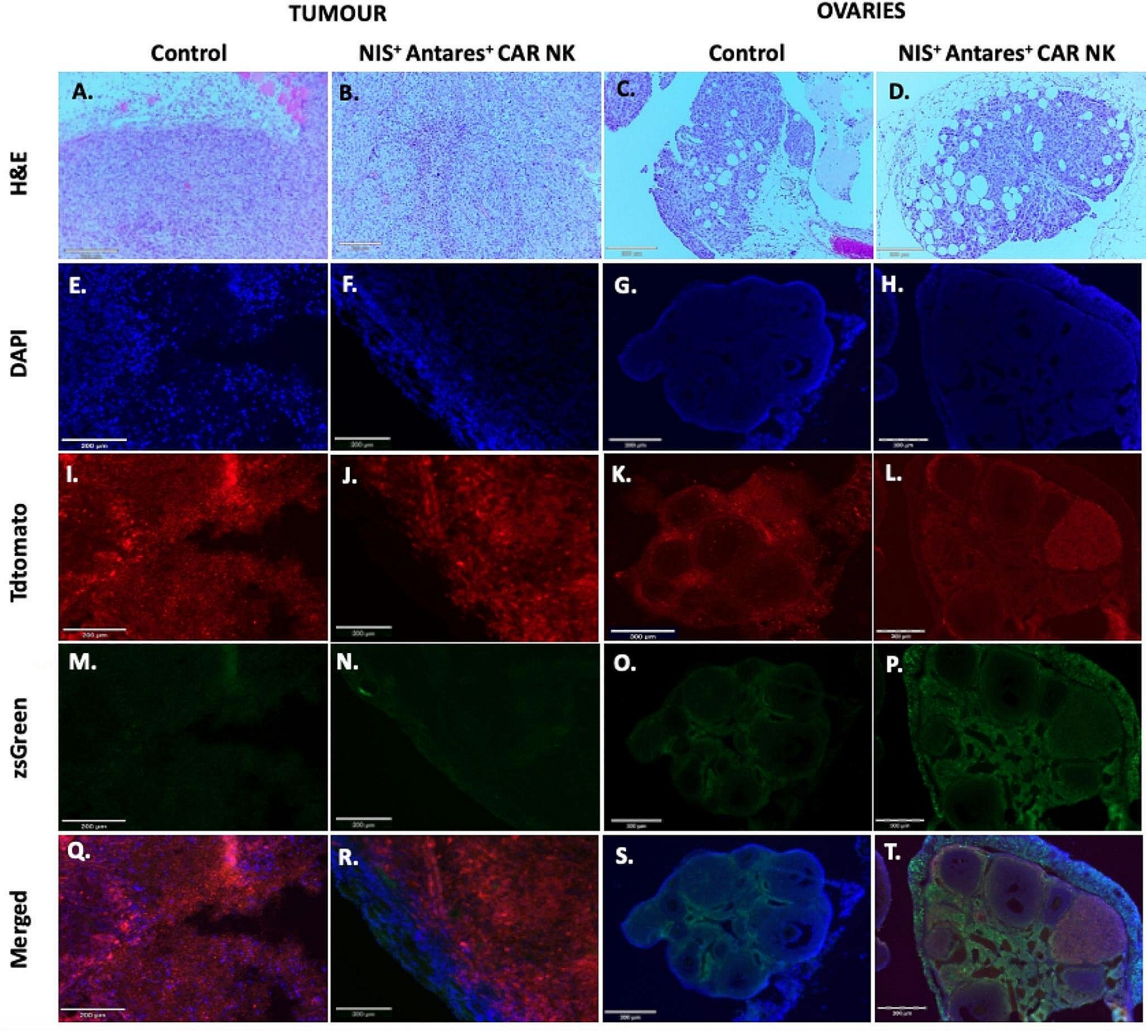
Tissue sections of tumor masses (**A-B**) and ovaries (**C-D**) stained with Hematoxylin and Eosin (**H**&**E**) of a control mouse and a mouse receiving the NIS^+^ Antares^+^ CAR NK therapy. Fluorescence images showing DAPI (nuclei; **E-H**), TdTomato (Fluc^+^tdT^+^SKOV3-ip1 cells; **I-L**), zsGreen (NIS^+^ Antares^+^ CAR NK; **M-P**), and the merged images (**Q-T**). ZsGreen positive cells are shown on the periphery of the ovaries in mice receiving the NK therapy (**P** and **T**). Few zsG positive cells are seen infiltrating inside the ovaries

At endpoint, tumors and ovaries were harvested and preserved for H&E staining on paraffin sections, as well as fluorescence microscopy of frozen sections (Fig. 5). Figure 5 shows representative sections of tumors and ovaries in mice receiving either PBS injections or NIS^+^ Antares^+^ CAR NK cell therapy.

TdTomato fluorescence (from Fluc^+^tdT^+^SKOV3-ip1 cells) is present in tumors from both control mice and mice receiving NIS^+^ Antares^+^ CAR NK cells. Minimal tdTomato fluorescence is observed in the ovaries of both cohorts of mice. Mice receiving NIS^+^ Antares^+^ CAR NK cells (also zsG-positive) showed no evidence of zsG fluorescent cells within the main tumor masses at endpoint (Fig. 5N). Instead, we noted many zsG fluorescent cells were surrounding the ovaries (Fig. 5P), potentially identifying these cells as the source of the distinct spherical PET signals within the peritoneum seen in Fig. 3D.

## Discussion

Clinical use of CAR T cell therapies for the treatment of leukemia, lymphoma and multiple myeloma has shown extraordinary outcomes for some patients with relapsed/refractory disease [59–62]. However, not all patients respond to this treatment, and many can relapse. CAR T cells are also associated with significant safety issues including cytokine release syndrome (CRS) and neurotoxicity [63, 64]. So far, the therapeutic benefit of this therapy for solid tumors has been less encouraging, leading to a belief that this therapy is perhaps unsuitable for solid tumors [65–67]. However, recent remarkable successes in patients with diffuse midline gliomas and neuroblastoma using CAR T cells targeting the disialoganglioside GD2 are beginning to provide hope [68].

CAR NK cells are becoming an attractive alternative to CAR T cells due to their high safety profile. CAR NK therapies can circumvent CRS and graft versus host disease (GvHD). They can also provide an “off the shelf” allogenic product and elicit anti-tumor effects in CAR-independent mechanisms through intrinsic activation receptors as well as via anti-body dependant cell-mediated cytotoxicity (ADCC) [28, 69].

Imaging tools that allow post-infusion tracking of these therapeutic cells could provide essential information such as sensitive and real-time tracking of cellular therapies, the potential to predict side effects due to rapid and uncontrolled proliferation, as well as predict therapeutic outcome, or lack thereof. In this study we develop a HER2 targeting-CAR NK therapy which can be visualized with the PET reporter gene, the Sodium-Iodide Symporter (NIS). Furthermore, CAR-NK cells were additionally engineered with Antares, which serves as a BLI reporter gene for sensitive *in-vivo* cellular tracking when administered with its optimized substrate fluorofurimazine (FFZ) [70].

Several PET reporter genes have been used to track CAR-T cells such as herpes simplex virus-thymidine kinase (HSV1tk), *Escherichia coli* dihydrofolate reductase enzyme (eDHFR), somatostatin receptor 2 (SSTR2) and prostate-specific membrane antigen (PSMA) [47–49, 71]. Due to their non-human origin, eDHFR and HSV1tk and variants have shown immunogenicity [72]. Furthermore, HSVtk-expressing CAR T cells were shown to be less effective in tumor killing than CAR T cells alone, and increased HSVtk expression affected CAR T cell viability [49]. While other reporter genes, such as SSRT and PSMA are human-derived and would pose minimal immunogenicity, SSRT-expressing CAR T cells have been reported to require tracers (i.e. ^68^Ga-DOTANOC, ^68^Ga-DOTATOC, ^68^Ga-DOTATATE) which activate T cells and PSMA-expressing CAR T cells have shown potential issues with respect to PSMA overexpression [72]. We employed NIS as our PET reporter gene as NIS is human-derived with minimal immunogenicity that is uniquely characterized by its ability to rapidly uptake radiotracers, with the majority of its tracer up taken in the first 10 min post administration [73]. It is also compatible with a clinical fluorine-based PET tracer, ^18^F-tetrafluoraborate (^18^F-[TFB]) whose radionuclide has a short half-life (110 min), short positron diffusion range in tissue (< 2.4 mm), and high positron yield [74], to provide a sensitive approach for PET whole body imaging. Several groups have utilized the advantages of NIS for imaging of various immune cells [72, 75, 76]. Lee et al., first employed NIS to track migration of dendritic cells (DC) to lymph nodes with both ^124^I and ^18^F[TFB] for PET/CT detection [77]. Similarly, Emami-Shahri’s et al., has also developed NIS-expressing CAR T cells with administration of [^99m^Tc]TcO4^−^ radiotracer for SPECT/CT imaging [75]. To our knowledge, this is the first time NIS-expressing CAR-NK cells have been imaged with PET.

Our in vitro studies showed that CAR NK cells with or without NIS reporter gene showed similar levels of cytotoxicity towards SKOV3-ip1 cells, indicating NIS expression did not influence CAR lytic function. The addition of a CAR component increased cytotoxicity above naïve NK-92 cell killing (Fig. 1). As performed previously [58, 78], we employed dual in vivo BLI to monitor both cancer cell and CAR NK cell populations in the same animals over time. Firefly luciferase (Fluc) was used to monitor SKOV3-ip1 cells, and we show mice receiving either PBS, or naïve NK injections showed significantly higher tumor burden when compared with mice receiving the NIS^+^ Antares^+^ CAR NK cell treatment (Fig. 2B). Thus, our HER2-targeted CAR-NK cells were able to significantly slow tumor progression in this highly aggressive model of HER2 ovarian cancer and prolonged the overall survival of treated mice (Fig. 2C). Although the HER2 CAR therapy did not cure the mice, future studies can look at optimizing CAR construct, increase CAR expression levels, or increase the dose of administered CAR NK therapy.

To monitor the CAR-NK cell population, we used the BLI reporter Antares, which is an optimized fusion of two orange-red fluorescent proteins called CyOFP1 excited by cyan light with NanoLuc [70]. Antares is a highly sensitive BLI reporter which can produce drastically brighter in vivo signal from deep tissues compared to FLuc and other BLI reporters. We also employed the Antares substrate FFZ which has been shown to strongly enhance bioluminescence signal in vivo, in comparison to other Antares-compatible substrates, to maximize sensitivity towards CAR NK detection [58]. Our Antares BLI data showed signal from CAR-NK cells present within the peritoneal spaces of mice, indicating the co-localization of both immunotherapy and tumor cells. NIS^+^ Antares^+^ CAR NK cells were longitudinally imaged using BLI with a stronger BLI signal originating from the bottom right quadrant of the animal (Fig. 3A.). This area also corresponds to the location of a large tumor mass observed in this and other sacrificed animals after exposure of their peritonea (Fig. 4A.), suggesting localized proliferation of therapeutic NK cells at the sites of tumors. To further validate this system in vivo, non-invasive imaging of the NIS-expressing CAR NK cells was performed. While uptake was observed in endogenous tissues as expected, mice receiving the NIS^+^ Antares^+^ CAR NK cell therapy also displayed uptake in other sites within the intraperitoneal cavity (Fig. 3D, blue arrows). No uptake was observed in the right quadrant as was seen with Antares BLI which could indicate the lack of NIS^+^ Antares^+^ CAR NK cells present at the time of PET imaging (Day 27). Upon histological comparisons, no zsGreen fluorescence was observed at the primary tumor sites, matching the lack of PET uptake in those regions.

NK-92 cells are an Il-2 dependent cell line. While some studies have irradiated the NK-92 cells prior to implantation to prevent in vivo expansion, others have provided Il-2 without irradiation [35]. Similarly, studies have engineered NK-92 cells to endogenously express Il-2 and other cytokines (i.e. Il-15) [79, 80]. In our study, after day 33 IL-2 injections were halted to suppress the growth and expansion of NK cells in vivo. As seen in Fig. 4B, Antares BLI signal showed stable levels until endpoint. In the future we will explore co-engineering our NK-92 cells with a suicide gene such as the Herpes simplex virus- thymidine kinase (HSV-tk) or human induced Caspase 9 (iCas9), to allow their selective killing following a defined treatment period [81, 82]. Interestingly, NIS may also be explored as a suicide gene by administering mice with Iodide-131, which would limit the size of our lentiviral constructs [83–85]. However, this approach would result in cytotoxicity in NIS-expressing endogenous tissue (thyroid, stomach, salivary glands, and mammary glands).

After an average peak Antares BLI signal on day 25, PET was performed on day 27. Mice receiving either PBS or NIS^+^ Antares^+^ CAR NK cell injections were imaged with PET post IV injections of ^18^F-[TFB]. Assessment of SUV revealed significantly higher (*p* < 0.05) uptake in mice receiving NIS^+^ Antares^+^ CAR NK cells compared to mice receiving PBS injections in areas within the peritoneal space (Fig. 3E.). Although stand-alone PET is limited in providing anatomical context, it is believed that the increased SUV in the peritoneal spaces (in Fig. 3D blue arrows) corresponds to the ovaries as seen in Fig. 5J., indicating homing of NK cells to the ovaries in mice receiving the NIS^+^ Antares^+^ CAR NK treatment.

Peritoneal washes with PBS were performed on day 30 to collect cells from within the peritoneal space and assess for either the absence or presence of zsGreen positive cells in one mouse receiving PBS and another receiving NIS^+^ Antares^+^ CAR NK cells. As shown in Fig. 4C, the cell aspirate showed approximately 3% of cells to be tdTomato positive, indicating the presence of SKOV3-ip1 cells. The peritoneal wash from the mouse receiving NIS^+^ Antares^+^ CAR NK cell injections show approximately 2% of cells expressing tdTomato and approximately 1% of cells were zsGreen positive (Fig. 4E), indicating the presence of both SKOV3-ip1 and NIS^+^ Antares^+^ CAR NK cells. Upon exposure of peritoneal spaces of mice, a larger tumor mass, in addition to ascites along the inner peritoneal lining, were evident in the control mouse compared to the mouse from the treatment group (Fig. 4B &D.). Thus, while this therapy did not eliminate the entirety of tumor masses, it showed robust tumor lysis and could be used as an adjuvant therapy, post standard chemotherapy or surgical debulking.

Histological sections and tissue fluorescence of the ovaries and tumor sections were observed. Sections of ovaries in mice receiving sham injections show similar DAPI and tdTomato to mice receiving the NIS^+^ Antares^+^ CAR NK cell therapy. Mice receiving the NK therapy show additional zsGreen fluorescence along the cortex of the ovaries, indicating some peripheral infiltration of the therapy. Sections of tumor masses for mice receiving the sham injections as well as mice receiving the NIS^+^ Antares^+^ CAR NK cell therapy both show qualitatively similar DAPI and tdTomato fluorescence. Mice receiving the NIS^+^ Antares^+^ CAR NK cells therapy do not show zsGreen fluorescence within their tumor mass at the time of sacrifice. Fluorescence sections of mice receiving NK therapy also imply that tumor infiltration is still a challenge for this therapy and methods to improve tumor penetration are needed. Furthermore, the incomplete eradication of the tumor in mice receiving the NIS^+^ Antares^+^ CAR NK cell therapy could indicate insufficient numbers of therapeutic cells (either at time of administration or their in vivo persistence).

### Limitations

While NIS^+^ Antares^+^ CAR NK cell therapy showed effective antitumor activity, complete tumor clearance was not shown. To improve tumor lysis, enhancements to the CAR can be incorporated such as using more potent generation CARs which include intrinsic cytokine release from NK-92 cells to alleviate dependence on exogenous cytokines, or incorporate multiple tumor antigen recognition domains to overcome antigen escape [12]. There are also limitations associated with the imaging platforms used for tracking the NIS^+^ Antares^+^ CAR NK cells. One of the limitations of the NIS reporter gene imaging is its background expression in organs such as the thyroid, stomach, and salivary glands, in addition to urinary clearance. This poses a challenge to visualize cell trafficking to these areas. To further exacerbate this limitation, PET lacks sufficient anatomical information to provide accurate distribution information on the cells. While this was not a major challenge for our peritoneal ovarian cancer model, it may restrict the use of this imaging reporter gene for other cancers with different biodistribution and metastatic spread. Another limitation with this cell theranostic system is the genetic incorporation of the CAR as well as the reporter genes using lentiviral vehicles, which poses clinical challenges. Recently, black box warnings have been added to some CAR-T therapies due to the emergence of rare cases where patients have developed T cell leukemia, presumably caused by lentiviral-driven random integration of the CAR construct into genome [86]. Further optimization of this cell system can be accomplished using genome editing technologies such as CRISPR, which would improve the safety by enabling site-selective integration into genome as opposed to the random integration [87]. Notably, the peritoneal lavage was only performed on one mouse per group which is insufficient to make independent conclusions, rather it was used to contribute or suggest conclusions.

## Conclusion

To conclude, the natural killer cell line, NK-92 cells, were genetically enhanced to incorporate a HER2 targeting CAR for treatment of HER2 positive ovarian cancer. Moreover, HER2-CAR NK cells were transduced to incorporate a BLI and PET reporter gene to accomplish non-invasive cell tracking with both imaging modalities in ovarian cancer bearing mice. The NIS reporter system provides a clinically compatible, safe, quantitative, platform to non-invasively visualize CAR NK cells using ^18^F-TFB-PET. With continued development this imaging has the potential to provide clinicians with missing information on the number, localization, viability, therapeutic efficacy as well as side effects of the immunotherapeutic cells. This comprehensive information could help clinicians better understand how women will respond to this therapy, as well as manage or intervene at earlier time points, to alleviate potential side-effects.

## Data Availability

Not applicable.
